# The impact of age on stress urinary incontinence outcomes in patients who underwent trans-obturator tape procedures

**DOI:** 10.12669/pjms.41.6.9793

**Published:** 2025-06

**Authors:** Bekir Kahveci, Yusuf Ziya Kizildemir

**Affiliations:** 1 Bekir Kahveci , Department of Obstetrics and Gynecology, Sanliurfa Training and Research Hospital, Sanliurfa, Turkey; 2 Yusuf Ziya Kizildemir, Department of Obstetrics and Gynecology, Sanliurfa Training and Research Hospital, Sanliurfa, Turkey

**Keywords:** Age, Continence surgery, Stress urinary incontinence, Trans-obturator tape

## Abstract

**Objective:**

This study aimed to investigate the effect of age on stress urinary incontinence outcomes in patients undergoing the outside-in trans-obturator tape (TOT) procedure.

**Methods:**

A retrospective cohort study was conducted on patients who underwent the outside-in TOT procedure from August 2017 to July 2023 at the Sanlıurfa Training and Research Hospital. We separated patients into two groups according to age: Group-I ≤ 50, Group-II > 50. The Patient Satisfaction Questionnaire and International Consultation on Incontinence Questionnaire—Short Form (ICIQ-SF) were administered. The primary outcomes were the post-operative recovery rate and the post-operative ICIQ-SF score.

**Results:**

Seventy-seven patients who underwent outside-in TOT surgery, of which 45 (58.4%) patients were in Group-I, and 32 (41.6%) patients were in Group-II. Surgical outcomes were evaluated; while cured, improved and failed outcomes were observed in 26 (57.8%), 16 (35.6%) and 3 (6.7%) patients in Group-I, these values were 13 (40.6%), 12 (37.5%) and 7 (21.9%) patients in the Group-II (p = 0.105). Similarly, the number of patients who were satisfied, moderately satisfied and dissatisfied with the procedure were 28 (62.2%), 13 (28.9%) and 4 (8.9%) in Group-I, while these values were 14 (43.8%), 11 (34.4%) and 7 (21.9%) in Group-II (p = 0.085). The median post-operative ICIQ-SF score was statistically significantly higher in Group-II (1 vs 7.5, p = 0.003).

**Conclusions:**

Although the treatment success and patient satisfaction with the TOT surgery were relatively high in the younger age group, the difference was not significant. In addition, the post-operative ICIQ-SF score was significantly higher in the older age group.

## INTRODUCTION

Stress urinary incontinence (SUI), defined as involuntary urinary leakage that occurs with increased intra-abdominal pressure such as coughing, sneezing or exercise, affects 12% to 46% of women.^1^ It is not only associated with significant physical morbidity, sexual dysfunction and reduced quality of life, but it is also of significant economic importance in terms of healthcare.^2^ Management options for SUI include conservative and surgical treatments. The outside-in trans-obturator tape (TOT) procedure was improved by Delorme in 2001 to prevent complications from blind passage in the retropubic space and to prevent major injuries.^3^ TOT is a less invasive surgical treatment option for SUI; it requires less dissection, causes less pain, provides faster patient mobilisation and requires a shorter hospital stay than previous open or laparoscopic surgeries.^4^ A recent review demonstrated that midurethral slings (MUS) are the most effective and safest surgical procedures for the management of SUI.^5^

Urinary dysfunctions in the elderly has been attributed to multiple factors, including detrusor overactivity, decreased bladder capacity and increased bladder sensation. Abrar et al.^6^ found that the frequency of SUI increased in pregnant women, especially those over 35 years of age. SUI is often associated with weakened supporting tissues, which creates an anatomical defect that causes the hypermobility of the bladder outlet and urethra. For these reasons, the prevalence of SUI increases with age.^7^ TOT as a surgical treatment for SUI has been found to be effective and safe amongst different age groups.^4^ However, according to some research, higher age at surgery and higher body mass index (BMI) have a detrimental effect on the objective cure rate five years after MUS surgery. It has also been found that higher age exerts a negative effect on subjective long-term outcomes.^8^ For the increasing population of elderly patients with incontinence, we need to evaluate whether the TOT procedure can be performed safely and effectively in older women.

A small number of published studies have compared treatment outcomes between different ages, and it is controversial whether advanced age influences post-surgical outcomes for SUI.^9^ Therefore, the aim of this study was to evaluate the effect of age on the efficacy and safety of the outside-in TOT procedure.

## METHODS

A retrospective cohort study was conducted on patients who underwent the outside-in TOT procedure from August 2017 to July 2023 at the Sanlıurfa Training and Research Hospital, in accordance with the Helsinki protocol .

### Ethical Approval:

It was obtained from the ethics committee. (Number: 21.03.2022/HRÜ.22/06/05; dated March 21, 2022).

The data used in this study were obtained from the patients’ computer and file records, telephone interviews, physical examinations and interviews during follow-up visits. Women undergoing primary outside-in TOT were separated into two groups according to age: Group-I ≤ 50 and Group-II > 50. The inclusion criteria required patients who received outside-in TOT for SUI without needing concurrent procedures. Exclusion criteria were applicable to patients with a history of incontinence surgery and radical pelvic surgery, detrusor overactivity, concomitant pelvic organ prolapse repair and post-operative follow-up of less than six months. All patients were evaluated routinely at least one year after surgery. The patients were called by phone, and the Patient Satisfaction Questionnaire (PSQ) and International Consultation on Incontinence Questionnaire-Short Form (ICIQ-SF) were administered. ICIQ-SF scores were calculated by asking the patients ‘How often do you leak urine?’, ‘We would like to know how much urine you think leaks. How much urine do you usually leak (whether you wear protection or not)?’ and ‘Overall, how much does leaking urine interfere with your everyday life?’.^10^ The PSQ consists of a single question, namely, ‘How satisfied are you with your progress since your surgery?’, for which three responses are possible: ‘satisfied’, ‘moderately satisfied’ or ‘dissatisfied’.^11^

Demographics (including age, BMI, parity, previous cesarean section, smoking status and experience of menopause), clinical features (including comorbidity or diabetes mellitus, hypertension and hyperlipidaemia) and outcomes were obtained. The primary outcomes were the post-operative recovery rate (cured, improved or failed) and post-operative ICIQ-SF score. Patients were identified as ‘cured’ when they had no recurrence of persistent urinary leakage symptoms after surgery. Improvement was identified as a significant decline in self-reported urine leakage without further treatment and no leakage during a stress test. Failure was identified as persistent or recurrent SUI. Secondary outcomes included patient satisfaction (satisfied, moderately satisfied or dissatisfied), duration of hospital stay, operative time, blood loss, post-operative ICIQ-SF score and complication rate (voiding difficulty, vaginal erosion, vaginal perforation, de novo urgency, operative site bleeding, urinary retention, suprapubic/thigh pain and dyspareunia).

**Fig.1:**
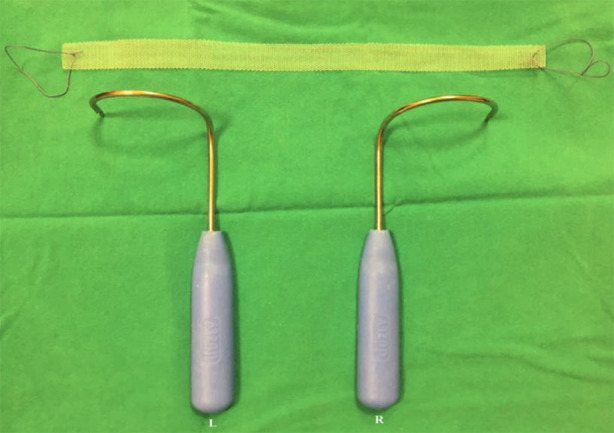
The appropriate size of polypropylene mesh and right - left TOT needle.

**Fig.2:**
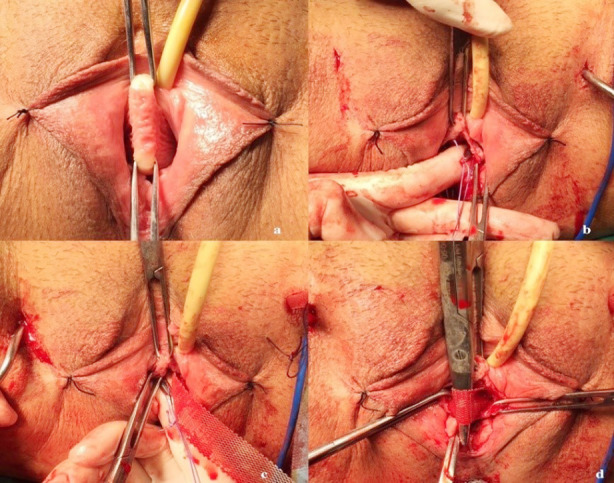
(a): Midurethral vaginal incision. b. Dissecting the tract for the trocar and mesh. c. Making groin incisions and inserting the trocars. d. Adjusting sling tension and closure of incisions (shared with the patient’s consent).

**Fig.3:**
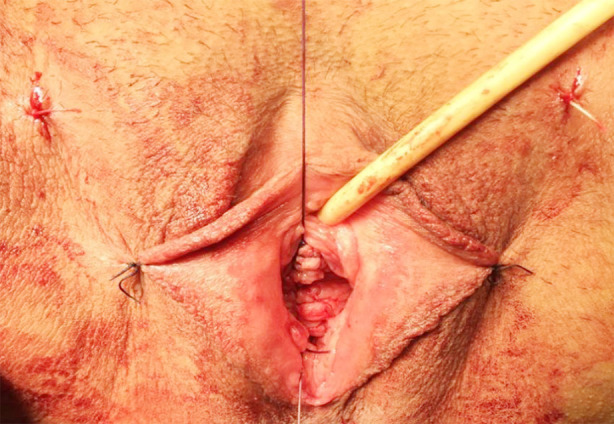
Final image of the midline incision closed with 2-0 vicryl locking suture technique.

TOT surgery was performed on all patients using the outside-in trans-obturator technique. During the surgical preparation, the appropriate size of the polypropylene mesh was cut, and ring sutures were placed at both ends of the mesh. Right and left TOT needles were then selected according to pelvic size (Fig.1). A sharp incision was made in the vaginal epithelium starting 1 cm proximal to the urethral meatus in the midline and extending 2 to 3 cm upwards (Fig.2A). Submucosal tunnels were created under the vaginal epithelium on both sides of the urethra using Metzenbaum scissors and blunt finger dissection. These tunnels were advanced upwards and behind the iliopubic rami (Fig.2B). The two planned groin exit points were marked at the level of the clitoris, 1-2 cm lateral to the genitofemoral folds. The TOT needle tip was inserted into the inguinal incision and advanced upward until the obturator membrane was pierced, and this transition was felt (popping sensation). The finger in the vagina was inserted into the ipsilateral vaginal tunnel and positioned upwards and behind the pubic arm. Using the inclination of the TOT needle, the tip of the needle was directed to the fingertip, and the needle was delivered to the vagina (Fig.2C). Metzenbaum scissors were placed between the urethra and the mesh to act as a space and to take the tension of the mesh by creating a gap (Fig.2D). Finally, the midline incision was closed with a 2-0 vicryl locked suture technique; the groin incision entry points were closed with a suture, and a tampon was placed in the vagina (Fig.3). Patients who were found to be able to urinate comfortably when the tampon was removed 24 hours after surgery were discharged.

### Statistical analysis:

The statistical significance of nominal variables between the two methods was tested with chi-square, and continuous variables were tested with the independent T-test and the Mann–Whitney U test. In all statistical analyses, the significance level was accepted as p < 0.05, and IBM SPSS 25 (IBM Corp, Armonk, NY, USA) software was used.

## RESULTS

A total of 77 patients were included, comprising 45 (58.4%) patients aged less than or equal to 50 years and 32 (41.6%) patients over 50 years old with SUI who underwent outside-in TOT surgery. The mean age was 42.8 ± 4.5 years for the young group and 58.7 ± 7.5 years for the older group. The presence of comorbidity, which was mostly accompanied by hypertension (52.0%), was statistically significant between groups 1 and 2 (22.2% vs 46.9%, p = 0.021). Percentages who had undergone previous cesarean sections (37.8% vs 21.9%) and with a history of smoking (26.7% vs 34.4%) were different between the groups, but this difference was not statistically significant (p = 0.108 and p = 0.316, respectively), (Table-I). At a minimum of six months, a complete cure was seen in 39 (50.6%) cases, while improvement was seen in 28 (36.4%) cases, with 10 cases (13%) failing.

**Table-I:** Demographic and clinical characteristics of the groups.

Variables	Group-I (n = 45)	Group-II (n = 32)	P-value
Age (year) ^†^	42.8 ± 4.5	58.7 ± 7.5	0.001*
BMI (kg/m^2^) ^†^	28.3 ± 4.2	27.0 ± 3.5	0.140
Parity ^‡^	4 (2-9)	5 (3-12)	0.076
Comorbidity^§^	10 (22.2)	15 (46.9)	0.021*
Diabetes mellitus	4 (8.9)	5 (15.6)	
Hypertension	5(11.1)	8 (25.0)	
Hyperlipidaemia	1 (2.2)	2 (6.3)	
Previous cesarean section^§^	17 (37.8)	7 (21.9)	0.108
Smoker^§^	12 (26.7)	11 (34.4)	0.316
Menopausal^§^	2 (4.4)	31 (96.9)	0.001*

BMI, body mass index; Group-I, age ≤ 50 years; Group-II, age > 50 years.

† Data are presented as mean ± standard deviation.

‡ Data are presented as median (range).

§ Data are presented as number (percentage).

* P ≤ 0.05 is statistically significant.

The surgical outcomes were evaluated (Table-II): while cured, improved and failed surgeries were observed in 26 (57.8%), 16 (35.6%) and 3 (6.7%) patients in Group-I, these values were 13 (40.6%), 12 (37.5%) and 7 (21.9%) patients in Group-II (p = 0.105, respectively). Although the difference was not statistically significant, the overall rate of cure + improvement was higher in the younger age group than the older age group (93.3% vs 78.1%). Similarly, the patients who felt satisfied, moderately satisfied and dissatisfied with the procedure were calculated at 28 (62.2%), 13 (28.9%) and 4 (8.9%) in Group-I and 14 (43.8%), 11 (34.4%) and 7 (21.9%) in Group-II (p = 0.085, respectively). The median hospital stays (1.3 ± 0.5 days vs 1.6 ± 0.7 days) and operative times (54.2 ± 13.0 min vs 61.3 ± 18.9 min) were higher in Group-II (p = 0.031 and p = 0.076). The median post-operative ICIQ-SF score was statistically significantly higher in Group-II (1 vs 7.5, p = 0.003).

**Table-II:** Surgical outcomes of TOT procedure.

Variables	Group-I (n = 45)	Group-II (n = 32)	P-value
***Clinical outcome^†^***			0.105
Cured	26 (57.8)	13 (40.6)
Improved	16 (35.6)	12 (37.5)
Failed	3 (6.7)	7 (21.9)
Cured + improved	42 (93.3)	25 (78.1)
***Patient satisfaction^†^***			0.085
Satisfied	28 (62.2)	14 (43.8)	
Moderately satisfied	13 (28.9)	11 (34.4)	
Dissatisfied	4 (8.9)	7 (21.9)	
Hospital stay (days)^‡^	1.3 ± 0.5	1.6 ± 0.7	0.031*
Operative time (min) ^‡^	54.2 ± 13.0	61.3 ± 18.9	0.076
Blood loss (cc)^‡^	22.8 ± 6.5	22.7 ± 6.7	0.967
Post-operative ICIQ-SF score^§^	1 (0-16)	7.5 (0-21)	0.003*

ICIQ-SF, International Consultation on Incontinence Questionnaire—Short Form; Group-I, age ≤ 50 years; Group-II, age > 50 years.

† Data are presented as number (percentage).

‡ Data are presented as mean ± standard deviation.

§ Data are presented as median (range).

* P ≤ 0.05 is statistically significant.

**Fig.4:**
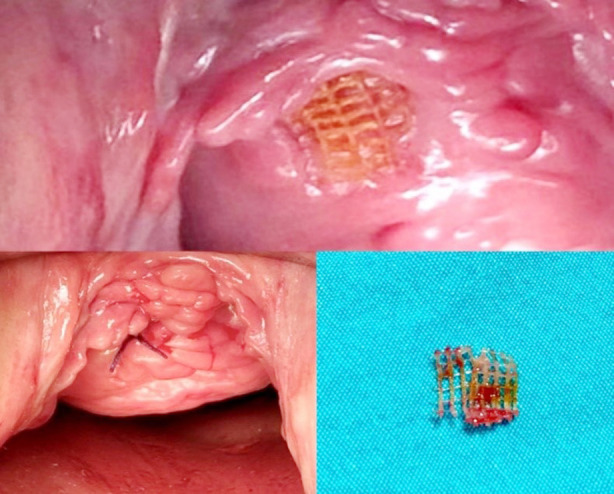
Surgical repair of mesh exposure.

**Fig.5:**
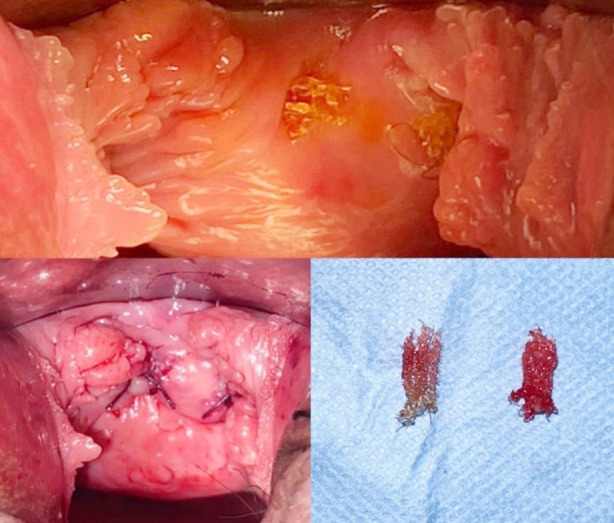
Surgical repair of the ruptured mesh.

While the total complication rate was 42.8% (33 patients) of the entire study, the same rate was 37.8% (17 patients) in Group-I and 50% (16 patients) in Group-II (p = 0.286). Similar numbers of complications were observed in the two groups, with de novo urgency (14.3%) and suprapubic/thigh pain (11.7%) being the most common. Vaginal erosion occurred for 1 (2.2%) patient in Group-I and 2 (3.1%) patients in Group-II (Table-III). This was observed in patients one year after surgery. All patients with epithelial erosion were treated with local excisions and local oestrogen therapy. Surgical repair of partial mesh exposure, also known as erosion, is depicted in Fig.4 and Fig.5.

**Table-III:** Post-operative morbidity of TOT procedure.

Variables	Total (n = 77)	Group-I (n = 45)	Group-II (n = 32)	P-value
Complication rate^†^	33 (42.8)	17 (37.8)	16 (50)	0.286
Voiding difficulty	4 (5.2)	2 (4.4)	2 (6.3)	
Vaginal erosion	3 (3.9)	1 (2.2)	2 (6.3)	
Vaginal perforation	1 (1.3)	1 (2.2)	0 (0.0)	
De novo urgency	11 (14.3)	7 (15.6)	4 (12.5)	
Operative site bleeding	2 (2.6)	1 (2.2)	1 (3.1)	
Urinary retention	1 (1.3)	0 (0)	1 (3.1)	
Suprapubic/thigh pain	9 (11.7)	5 (11.1)	4 (12.5)	
Dyspareunia	2 (2.6)	1 (2.2)	1 (3.1)	

Group-I, age ≤ 50 years; Group-II, age > 50 years.

† Data are presented as number (percentage).

*P ≤ 0.05 is statistically significant.

## DISCUSSION

Our study demonstrates that treatment success for and patient satisfaction with TOT as a surgical treatment for SUI were relatively higher in the younger age group. However, the difference was not statistically significant. In addition, the post-operative ICIQ-SF scores were significantly higher in the older age group, suggesting that post-operative complaints and failure rates may be higher in the older age group. Although the surgical risk in older women may be high due to the presence of comorbid conditions such as hypertension, diabetes mellitus, cardiac arrhythmias and hyperlipidaemia, the presence of any of these conditions should not be a contraindication to surgical procedures for urinary incontinence. Consistent with Yasa et al.,^12^ comorbidities were higher in women of older ages in our study. The study has demonstrated the short- and long-term efficacy and safety of TOT for the surgical treatment of SUI.^13^ In recent studies, a high recovery rate of 80% to 95.9% was found in patients who underwent TOT.^4^,^5^,^14^ According to a recent meta-analysis, TVT had a higher subjective cure rate than TOT, but TOT had a lower incidence of postoperative dyspareunia and vaginal mucosal perforation.^15^ However, in our study, the total cure rate was 50.6%, while improvement was 36.4%. Additionally, the patient satisfaction rate in our study was 54.5%, which is lower than what has previously been reported in the literature.

Ageing is responsible for the deterioration of the pelvic floor due to the decrease in the number of vascular plexuses in the urethra submucosa and the decrease in the collagen type I/III ratio, along with changes in the striated pelvic floor muscles. Laterza RM et al.^8^ demonstrated that higher age exertts a negative impact on objective and subjective long-term outcomes after MUS procedures. Likewise, Rechberger et al.^16^ emphasised that menopausal status and aging negatively affect surgical results. In another study of young, elderly and old women, the objective cure rates were 91.0%, 80.6% and 66.7%, and the subjective cure rates were 89.2%, 77.6% and 58.3%, respectively. It was found that cure rates decreased with age.^17^ However, Malek JM et al.^9^ emphasised that outcomes after MUS procedures do not differ between women under and over the age of 70. However, older women were found to have more persistent and urgent urinary incontinence, worse resolution of urinary tract symptoms and to be a risk factor.^5^ Yang JM et al. found that while older women were more likely to report bothersome SUI, the severity of SUI and postoperative adverse events were similar in older women compared with other age groups.^18^ Yasa et al.s^12^`s cure rates and patient satisfaction rates for older and younger patients after TOT surgery were similar in both groups. Similarly, in the current study, there was no significant difference between the older and younger patients in terms of clinical outcomes and patient satisfaction. Our data are consistent with previously published data by Ahn et al.,^4^ which indicated that the cure rate does not decrease with age.

A study of 100 patients undergoing TOT surgery with 3-D transperineal ultrasound found that a trans-obturator sling that moved in concert with the urethra during dynamic testing, was located in the mid-urethra and deformed appropriately with stress manoeuvres was associated with the best success rates.^19^ A recent study found that ultrasound revealed that slings tend to be looser and higher in older women.^18^ The optimal means to prevent the mid-urethral sling from being excessively proximal is to start the vaginal incision for the mid-urethral sling 1 cm from the urethral meatus and never use the same anterior incision for placement, as suggested in the current study. In the current study, de novo urgency was not different in the younger and older age groups and was found to be 14.3% in total. However, comorbidity was consistent with this study and was higher in older patients. Voiding dysfunction is commonly reported after midurethral sling placement. The reported rate of voiding dysfunction following trans-obturator sling procedures is 4% to 11%.^20^ In the current study, the voiding dysfunction rate was 5.2%.

Post-operative voiding dysfunction is typically managed initially with bladder catheterisation. Urinary retention that persists for four to six weeks may require a release of the sling. In one study of 205 women who underwent trans-obturator sling placement, for example, 1.5% required sling release or urethrolysis.^21^ In our study, we applied a bladder catheter and sling release to one patient in the older age group in the post-operative period. Even if the outlet obstruction identified in older women is temporary, they may have temporary retention after surgery but eventually regain normal urination. Groin pain occurs in approximately 12% to 16% of women following a trans-obturator sling procedure.^22^ Our study is consistent with the literature, with a rate of 11.7%. Dyspareunia has been reported in 1% to 9% of women following trans-obturator sling placement.^23^ In the current study, this rate was 2.6%.

We had three cases of partial mesh exposure (3.9%), which required mesh revision. This is similar to Zhang et al.’s^24^ (5.5%) and Abrar et al.’s 14 four cases (6.3%). We preferred local mesh excision in our study because in a case series of total mesh removal, 83% of patients developed incontinence that worsened significantly at follow-up after total mesh removal.^25^ To prevent vaginal erosion, a deep incision of Halban’s fascia in the midline of the vagina should be made along with careful closure of the vaginal wound. Vaginal perforation has been reported in 0.4% to 1.3% of women in studies.^26^,^27^ Our study is consistent with the literature and vaginal perforation was observed in one patient (1.3%). Perioperative vaginal perforation was noticed in this patient and the mesh was reinserted from the correct cleavage. It is also important to perform a proper sulcus dissection of the upper lateral vaginal wall to prevent vaginal perforation. The vaginal sulci should be inspected and palpated after the insertion of the trocars. Additionally, according to a recent meta-analysis, the incidence of vaginal mucosal perforation was lower in TOT than in TVT (OR, 95% CI = 0.11 (0.02, 0.61), p = 0.01).^15^

ICIQ-SF has been described as a useful international urinary incontinence questionnaire. Abrar et al.^6^ stated that the mean ICIQ-SF score corresponds to a severe impact on quality of life. Nyström et al.^10^ found that applying this form before and after SUI treatment is useful and reproducible. Sharma et al.^13^ found that the decrease in the ICIQ-SF score after surgery compared to before surgery was extremely significant. This revealed that TOT was highly effective in reducing SUI symptoms, with very high recovery rates. However, in the current study, we compared post-operative ICIQ-SF scores between younger and older patients. The scores were significantly higher in the older age group, which had a poor impression of improvement of their urinary tract condition.

### Strength of the study:

The strengths of our study includes the fact that the photographic images of TOT surgery are of high quality and educational and support current literature.

### Limitations:

The limitations of the study are that the number of patients is small and that it is retrospective in nature. To reach more definitive conclusions, prospective randomised controlled studies with more cases are needed in the future.

## CONCLUSION

Although the treatment success and patient satisfaction of TOT as a surgical treatment for SUI were relatively high in the younger age group, the difference was not statistically significant in this study. In addition, post-operative ICIQ-SF scores were significantly higher in the older age group, which had a worse impression regarding the improvement of their urinary tract condition. Furthermore, since there are few studies on this subject, we believe that the detailed explanation of the surgical steps and the results of this study, supported by photographs, will contribute to surgeons who will conduct research in this field.

### Authors Contributon:

***BK. YZK: Concept, design, literatdure search, critical review, analysis of data and interpretaitn. They have read the final version of the manuscript and are accountable for the integrity of the study*.**
